# Associations Between Human Milk Oligosaccharides and Maternal Nutrition: Latvian Study

**DOI:** 10.3390/nu18010136

**Published:** 2025-12-31

**Authors:** Līva Aumeistere, Kristīne Majore, Anete Keke, Annamarija Driksna, Svetlana Aleksejeva, Inga Ciprovica

**Affiliations:** Food Institute, Latvia University of Life Sciences and Technologies, Rīgas iela 22a, LV-3004 Jelgava, Latvia; liva.aumeistere@lbtu.lv (L.A.); kristine.majore@lbtu.lv (K.M.); anete.keke@lbtu.lv (A.K.); grava.annamarija@gmail.com (A.D.); s.aleksejeva@gmail.com (S.A.)

**Keywords:** human milk, human milk oligosaccharides, exclusive breastfeeding, diet

## Abstract

**Background/Objectives**: HMOs are the third most abundant solid component after lactose and fats in human milk. This study aimed to examine the relationships between maternal diet and HMO composition and concentration in human milk among lactating women in Latvia. **Methods**: Pooled 24 h human milk samples, 72 h food diaries, and questionnaires on anthropometric and sociodemographic characteristics were collected from 68 exclusively breastfeeding women residing in Latvia. HMOs were analyzed by UHPLC/FLD, and dietary data were analyzed using the Estonian NutriData program. **Results:** The eight most abundant HMO structures were determined with total concentration ranging between 178.66 and 32,910.09 mg L^−1^. 2′-FL was the most prevalent HMO in human milk (median concentration—3647 mg L^−1^), followed by 3′-FL (1436.74 mg L^−1^). Participants had an insufficient intake of vegetables, fruits, berries, milk and dairy products, and fish, leading to vitamin A, vitamin C, folate, and iodine intakes lower than recommended for lactating women. Limitation or exclusion of milk and dairy products from the diet was associated with a higher 2′-FL concentration in human milk (*p* = 0.037). Preference for “zero sugar” products was associated with a higher 3′-FL, 6′-GL, LNnT, 6′-SL, LNDFH II concentration in human milk (*p* < 0.050). Dietary supplement use (e.g., vitamin D, calcium) was also associated with differences in HMO composition and concentration in milk (*p* < 0.050). **Conclusions**: The findings highlight the importance of dietary habits and supplement use in shaping HMO profiles, though more human milk samples and dietary data need to be evaluated to draw further conclusions.

## 1. Introduction

Human milk is the gold standard for children during early life, recommended exclusively for the first six months and through the first two years as a key source of energy, nutrients, and immunological protection. Human milk consists of macro- and microelements, as well as bioactive compounds, which synergistically promote children’s immunity, growth, and well-being [1].

Among bioactive compounds in human milk, HMOs are the third most abundant solid component after lactose and fats. Human milk oligosaccharides derive from lactose, and specific HMO structures are built by elongation of N-acetyl-D-glucosamine (GlcNac), D-galactose (Gal), sialic acid (Neu5Ac), and L-fucose (Fuc) in a specific order and linkages to a lactose core [2].

The composition and concentration of individual HMO structures vary depending on genetic and non-genetic factors [3,4]. The presence of glycosyl transferases in the mammary gland is genetically determined for HMO biosynthesis, except for fucosyltransferases [5]; therefore, fucosylated HMO variability depends on the activity of fucosyltransferases encoded by the secretor and Lewis genes in lactating mothers [6,7,8,9].

Non-genetic factors that contribute to variation in HMO concentrations are the mother’s age, mental status [10], weight, body mass index [3,11], maternal diet, and different populations [12], although to a lesser extent than genetic factors. The effect of maternal diet as a non-genetic factor is still underexplored; only a few studies have analyzed the role of maternal diet [3,11,12,13,14] and probiotic supplementation on HMO patterns [15]. Therefore, more data are needed to confirm the relationship among a diverse range of study populations. Additionally, the published research data revealed significant differences in reported HMO concentrations [5] due to variations in methodologies and techniques for HMO analysis, as well as among individuals.

To date, no studies have been performed to analyze the most abundant HMOs in Latvian lactating mothers’ milk [16] in relation to maternal diet. The study aimed to develop the methodology of HMO analysis, evaluate the HMO profile in terms of non-genetic factor analysis, and examine the relationships between maternal diet and HMO composition and concentration.

## 2. Materials and Methods

### 2.1. Study Design and Selection of Participants

This study was conducted from 2024 to 2025 at the Food Institute of Latvia University of Life Sciences and Technologies. Human milk samples, food diaries, FFQ, participants’ sociodemographic and anthropometric data, and participants’ informed consents were collected after receiving approval from Rīga Stradiņš University Ethics Committee (2-PĒK-4/616/2024, approved on 21 October 2024).

The study was conducted according to the guidelines laid down in the following:

- Declaration of Helsinki [17];
- Convention for the Protection of Human Rights and Dignity of the Human Being concerning the Application of Biology and Medicine: Convention on Human Rights and Biomedicine [18];
- General Data Protection Regulation [19].

Written informed consent was received from all participants prior to the study. Participants were voluntarily recruited via invitations published on social networks. The inclusion criteria for the participants were as follows:

- At least 18 years old;
- Residing in Latvia;
- Singleton pregnancy;
- Term pregnancy (37–41 gestation weeks);
- Child’s birth weight ≥2500 g;
- At least 28 days postpartum;
- Mother exclusively breastfeeding;
- Infant no more than 6 months old;
- Woman currently nursing only one child;
- Mother and child healthy;
- Woman is not currently pregnant again;
- Signed consent form.

The exclusion criteria were as follows:

- Noncompliance with the inclusion criteria;
- Unsigned consent form.

### 2.2. Collection of Human Milk Samples, Dietary, Etc., Data

After the woman had completed the electronic informed consent form, the researcher contacted her via e-mail to agree on how to hand over the research materials—in person or via a self-service parcel machine (Omniva, Latvia). Research materials included the following:

- One polypropylene container, volume—50 mL, with a graduated mark for 40 mL (Plastiques Gosselin, France) marked with a unique four-digit code (participant number) for the collection of human milk sample;
- Human milk sampling instruction, 72 h food diary, FFQ, and questionnaire about anthropometric, sociodemographic, etc., questions in printed or electronic form.

Participants had to choose three consecutive days to fulfill the 72 h food diary and the questionnaires. No dietary restrictions were applied, and participants were able to consume a self-chosen diet. To make it easier to fill out the 72 h food diary, study participants had access to an online food atlas with portion sizes [20].

On the fourth day, participants had to collect a pooled 24 h human milk sample. A few milliliters of human milk were expressed after the end of nursing from the feeding breast. If the nursing session was paired, then from both breasts. Participants were able to choose the most convenient method for human milk collection (manual expression by hand or breast pump, or a combination of both methods). The sampling frequency was not specified, but considering potential diurnal variations, the pooled sample had to include milk from morning, midday, evening, and nighttime. Each participant was asked to collect at least 20 mL of human milk for the HMO analysis. During the sampling process, the collected human milk was kept in the refrigerator (~4 °C up to 24 h), and afterwards, a container with the pooled human milk sample was placed in the household freezer (approximately −18 °C). The frozen human milk samples were collected using a bag with ice packs and transported to the Food Institute of Latvia University of Life Sciences and Technologies (Jelgava, Latvia), where the chemical composition analysis and HMO were determined.

A total of 74 human milk samples were collected; however, only 68 of them were analyzed in relation to the associations between maternal nutrition and HMOs. Six participants did not submit the FFQ, 72 h food diaries, and/or questionnaires on anthropometric and sociodemographic characteristics; therefore, human milk samples were withdrawn from further analysis.

### 2.3. Evaluation of Dietary Data

The 72 h food diaries were analyzed using the NutriData program (Estonian National Institute for Health Development) [21]. Information about dietary supplements, meal replacements, and medication was taken from the manufacturers’ websites.

### 2.4. Human Milk Composition Analysis

The frozen milk samples were thawed before the proximate composition analysis and heated to 35 °C prior to analysis. Human milk fat, protein, lactose, and solids were determined using a milk analyzer MilkoScan^TM^ Mars (Foss, Hillerød, Denmark). Analyses were made in triplicate. After determination, human milk samples were frozen in a laboratory freezer and maintained in frozen conditions till HMO analysis.

### 2.5. Human Milk Oligosaccharide Analysis

The solvents used in the HMO analysis were acetonitrile (AC03782500; Scharlau, Barcelona, Spain), formic acid (AC10851000, Scharlau, Spain), ammonium formate (156264-500G, Sigma-Aldrich, St. Louis, MO, USA), glacial acetic acid (AC03461000, Scharlau, Spain), sodium hydroxide (221,465, purity ≥ 97.0%, Sigma-Aldrich, St. Louis, MO, USA), sodium cyanoborohydride (156,159, purity 95%, Sigma-Aldrich, St. Louis, MO, USA), dimethyl sulfoxide (34,869, purity ≥ 99.7%, Sigma-Aldrich, St. Louis, MO, USA), and distilled water.

The analytical standards used were 2′-Fucosyllactose (OF137553, min purity 90%, Biosynth, Compton, UK), 3-Fucosyllactose (SMB01365-25MG, purity ≥ 95%, Sigma-Aldrich, St. Louis, MO, USA), 6′-sialyllactose sodium salt (sc-221110, purity ≥ 98%, ChemCruz, Santa Cruz Biotechnology, Heidelberg, Germany), Lacto-N-difucohexaose I (OL01664, purity > 98%, Biosynth, UK), Lacto-N-difucohexaose II (OL06826, purity > 90%, Biosynth, UK), Lacto-N-neotetraose (OL05765, purity 95%, Biosynth, UK and GLY021-95%, purity > 95%, Elicityl, Crolles, France), 6′-Galactosyllactose (OG15070, purity > 95%, Biosynth, UK), 3′-Galactosyllactose (OG15069, purity > 95%, Biosynth, UK), 2-aminobenzamide (97,088, purity ≥ 98.0%, Supelco, Deisenhofen, Germany), and laminaritriose (O-LAM3, purity > 95%, Megazyme, Wicklow, Ireland).

Thawed samples were prepared for analysis, including lactose hydrolysis, protein precipitation, and centrifugation, according to ISO 7102:2024 [22].

Samples were purified and separated via HILIC on a Glycan BEH Amide column. During the sample analysis via FLD, a labelling reagent 2-aminobenzamide (0.35 mol L^−1^) and sodium cyanoborohydride (1.0 mol L^−1^) in dimethyl sulfoxide with 30% acetic acid was employed and subsequently separated using HILIC on a BEH Glycan column. A BEH Amide guard column preceded both columns, all of which were procured from Waters Corporation (Milford, MA, USA).

An amount of 100 μL human milk was mixed with the same volume of 2-aminobenzamide (0.35 mol L^−1^) and sodium cyanoborohydride (1.0 mol L^−1^) in dimethyl sulfoxide with 30% acetic acid) for a total of 200 μL. The solution was placed in a water bath at 65 °C for 1 h. After cooling for 5 min at 4 °C, 1000 μL of a 75% acetonitrile and 25% distilled water solution was added [23].

The labelled oligosaccharides were separated and quantified by UHPLC/FLD using a Shimadzu Nexera series LC-40 UHPLC system (Shimadzu, Tokyo, Japan) equipped with a fluorescence detector RF-10AXL (Shimadzu, Japan), equipped with ACQUITY UPLC BEH Glycan column (2.1 × 150 mm, 1.7 μm) (Waters, Milford, MA, USA) coupled to an ACQUITY UPLC BEH VanGuard™ Amide pre-column (2.1 × 5 mm, 1.7 μm) (Waters, Wexford, Ireland).

The injection volume of the samples was 10 μL, and the elution of the oligosaccharides was carried out using a gradient condition. The temperature of the analytical column was set to 60 °C. The mobile phase consisted of 50 mM ammonium formate (solvent A) and acetonitrile (solvent B). The gradient chromatographic condition is shown in Table 1.

The analytical system was validated using HMO standard curves, and repeatability was assessed with certain concentrations of a standard mix as a quality control to ensure accuracy across batches.

### 2.6. Statistical Analysis and Data Visualization

Data from questionnaires, 72 h food diaries, and human milk analysis were statistically analyzed using Microsoft Office Excel 360. Non-parametric tests, like Spearman’s rank correlation, independent-samples Mann–Whitney U test, and Kruskal–Wallis test, were applied to evaluate associations between HMO concentration and selected maternal and infant characteristics, as well as the impact of diet. These analyses were conducted using IBM SPSS Statistics 31. A *p*-value of ≤0.050 was considered statistically significant.

Data visualizations were generated using Microsoft Office Excel 360 and DataViz Kit [24].

## 3. Results

### 3.1. Characteristics of the Participants

Information about maternal and infant characteristics collected from the questionnaires is compiled in Table 2. The participants were from all statistical regions of Latvia (Riga, Vidzeme, Latgale, Zemgale, Kurzeme). Nevertheless, a substantial proportion of the participants were from the capital city, Riga (*n* = 30) or nearby municipalities within the Riga statistical region (*n* = 20).

Based on the participants’ reported anthropometrical data, two participants had a postpartum BMI classified as underweight (i.e., BMI < 18.5), 46 participants—normal weight (i.e., BMI between 18.5 and 24.9), 17 participants—overweight (i.e., BMI between 25.0 and 29.9), and three participants—obese (BMI ≥ 30.0).

None of the participants reported current smoking, but 12 participants reported smoking before pregnancy—heated tobacco products (*n* = 4) and/or e-cigarettes, vapes, and other electronic nicotine delivery systems (*n* = 7) and/or tobacco products for smoking—cigarettes, etc. (*n* = 3).

Sixteen participants reported that a family member was a smoker—using e-cigarettes, vapes, and other electronic nicotine delivery systems (*n* = 8) and/or using tobacco products for smoking—cigarettes, etc. (*n* = 8). One participant noted that she was not sure what type of device a family member was using for smoking.

### 3.2. Evaluation of Human Milk Composition

Prior to the analysis of HMOs, the composition of human milk was evaluated. The acquired results were consistent with average data reported in the literature (Table 3). Lactose, as the most abundant solid constituent of human milk, varied from 6.24% to 7.43% (median—6.99%), which is an increase of up to 7%, compared with our previously reported results [16].

The biosynthesis of HMOs begins with an elongated lactose molecule that is either fucosylated or sialylated, resulting in a diverse range of HMOs (Table 4).

**Table 4:** Human milk oligosaccharides, mg L^−1^ (*n* = 68).

HMO	This study, Median (Minimal–Maximal Value)	Other Studies, Median (Minimal–Maximal Value)
2′-FL	3647.05 (1.54–16,205.75)	1650 (7–4133) [26] 1700 (130–4800) [27] 2300 (690–4280) [4]
3′-FL	1436.74 (56.32–12,494.90)	543 (132–2013) [26] 730 (160–1900) [4] 1000 (50–3900) [27]
3′-GL	ND (ND–252.45)	4 (4–21) [26]
6′-GL	9.90 (ND–2473.90)	10 (10–40) [27] 15 (3–48) [26]
LNnT	291.39 (ND–1870.88)	30 (10–240) [27] 121 (27–285) [26] 310 (65–1240) [4]
6′-SL	132.06 (ND–947.71)	30 (10–290) [27] 192 (53–671) [26] 450 (0–740) [4]
LNDFH I	494.95 (ND–5511.00)	310 (20–990) [27] 749 (5–1935) [26] 1074 (5–2530) [4]
LNDFH II	146.08 (ND–779.79)	50 (20–240) [4]
Sum of HMOs	9730.46 (178.66–32,910.09)	4840 (3010–6590) [27] 10,288 (8600–16,790) [4]

**Figure 1:**
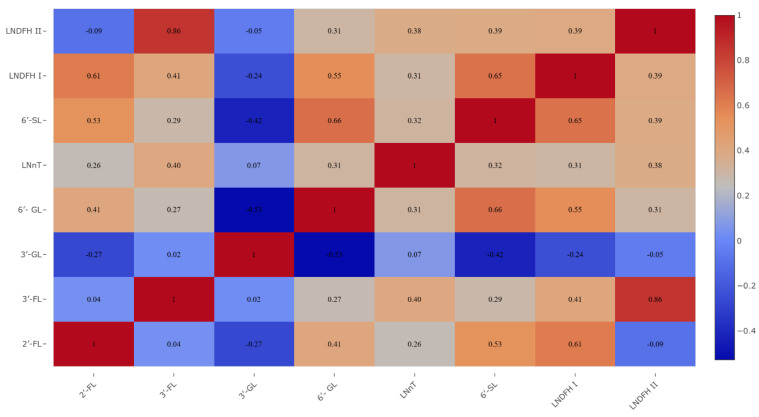
Correlations between individual HMOs in the tested human milk samples (*n* = 68).

In this study, the eight most abundant HMO structures were determined with an estimated total concentration ranging between 178.66 and 32,910.09 mg L^−1^. A small amount of galactosyl-lactoses (GLs), mainly 6′-GL and 3′-GL, were also found in the analyzed human milk samples. Eight participants (12%) had a 2′-FL concentration in human milk below 100 mg L^−1^, indicating that they were non-secretors. There were a few strong correlations observed between individual HMOs in the tested human milk samples (Figure 1).

There was a negative correlation between LNnT and fat, total solids concentration in human milk (ρ = −0.304, *p* = 0.012 and ρ = −0.288, *p* = 0.017, respectively).

Overall, maternal and infant characteristics were not observed to have a significant effect on HMO concentration (*p* > 0.050), except for 6′-SL and maternal age (ρ = −0.261, *p* = 0.032) and infant’s age (ρ = −0.403, *p* = 0.001). Smoking before pregnancy was linked to a significantly higher 6′-SL concentration in milk (234.36 mg L^−1^ compared to 112.39 mg L^−1^, *p* = 0.025).

### 3.3. Evaluation of Participants’ Dietary Habits

Information regarding participants’ dietary data is compiled in Table 5 and Table 6.

About one third of the participants (*n* = 23) reported eliminating or reducing certain foods in their diet, most frequently milk and dairy products (*n* = 17, 25%), to reduce health issues for the infant (cow’s milk protein allergy, colic, etc.). Most participants (*n* = 54) noted that they prefer whole grain products (whole grain bread, pasta, etc.) daily, while 13 participants reported that they prefer products in which sugar has been replaced with sweeteners (i.e., “zero sugar” products).

Median vegetable, fruit, and berry intake among participants did not reach the minimal daily amount (i.e., 500 g) recommended by the Nordic Nutrition Recommendations [28]. Based on the estimated median daily fish and seafood consumption (10.83 g), it is unlikely that participants’ intake would reach the recommended weekly intake of 300–450 g (ready-to-eat weight) [28]. Also, the participants’ median consumption of milk and dairy products (300.80 g per day) fell short of the dietary recommendations of 350–500 g per day [28].

**Table 5:** Product consumption among the participants, g per day (*n* = 68).

Food Groups	Median (Minimal–Maximal Value)
Cereal products—dry ingredients, pseudograins, corn products	79.69 (13.03–261.53)
Bread products	61.24 (0.00–238.47)
Potatoes	68.79 (0.00–282.63)
Vegetables, pulses, mushrooms	233.01 (32.00–932.62)
Fruits, berries	161.00 (0.00–991.00)
Milk, dairy products	300.80 (0.00–896.81)
Dairy product alternatives	0.00 (0.00–325.00)
Meat, meat products, offal	164.80 (0.00–508.87)
Fish, seafood	10.83 (0.00–217.40)
Eggs	44.67 (0.00–185.98)
Plant-based products used instead of meat, fish, eggs	0.00 (0.00–113.33)
Fats (butter, margarine, nuts, seeds, oleaginous fruits, etc.)	46.68 (4.29–186.84)
Sugar, honey, confectionery, dessert sauces	59.87 (0.00–335.67)
Pastry and bakery products	5.33 (0.00–296.67)
Savory snacks	0.00 (0.00–53.67)
Non-alcoholic beverages (water, coffee, tea, etc.)	2257.49 (658.33–4662.00)
Alcoholic beverages	0.00 (0.00–66.00)

Saturated fatty acid intake among the participants was higher than recommended (Table 6). It was mostly associated with milk and dairy product consumption (ρ = 0.455, *p* < 0.0005). Total carbohydrate intake was lower than recommended (i.e., less than 45 E%) [29]. Overall, a higher carbohydrate intake was observed among participants with a higher intake of cereal products, pseudocereals, corn products (ρ = 0.482, *p* < 0.0005), and fruits and berries (ρ = 0.442, *p* < 0.0005). Sodium intake was excessive and related to the intake of products from the food category—“Meat, meat products, offal” (ρ = 0.495, *p* < 0.0005). Insufficient vitamin A intake was associated with low vegetable consumption (ρ = 0.471, *p* < 0.0005).

**Table 6:** Energy and nutrient intakes among the participants (*n* = 68).

Energy, Nutrients	Median Intake (Minimal–Maximal Value)	Recommendations [29]
Energy (kcal)	2322.79 (1473.52–4864.51)	2393–3159
Protein (E%)	16.82 (9.72–33.60)	10–20 E% + 13–19 g
Fat (E%)	40.51 (22.72–57.87)	25–40
Saturated fatty acids (E%)	14.40 (3.36–24.91)	≤10
Monounsaturated fatty acids (E%)	14.58 (7.53–22.38)	10–20
Polyunsaturated fatty acids (E%)	7.18 (3.44–12.51)	5–10
Trans fatty acids (g)	0.67 (0.02–2.79)	as little as possible
Carbohydrate (E%)	41.56 (23.93–61.81)	45–60
Total sugar (g)	101.78 (45.19–262.05)	≤10 E% from added/free sugars (glucose, fructose, table sugar, as well as honey, syrups, fruit juice, and juice concentrates)
Sucrose (g)	47.99 (12.00–170.81)	
Lactose (g)	11.34 (0.00–41.16)	
Glucose (g)	15.51 (5.31–42.53)	
Maltose (g)	1.43 (0.08–14.23)	
Fructose (g)	17.17 (5.33–52.04)	
Galactose (g)	0.12 (0.00–7.69)	
Sugar alcohols (polyols) (g)	1.51 (0.02–9.51)	No recommendation provided
Dietary fiber (g)	27.47 (13.86–61.34)	≥25
Sodium (mg)	2547.26 (964.58–7561.81)	1500
Potassium (mg)	3618.12 (2058.21–6898.55)	3500
Calcium (mg)	1198.24 (377.16–2314.99)	950
Phosphorus (mg)	1630.80 (889.86–3432.63)	520
Magnesium (mg)	428.86 (198.26–883.75)	300
Iron (mg)	16.63 (4.95–78.45)	15
Zinc (mg)	15.66 (6.74–46.91)	13
Manganese (mg)	5.05 (1.83–28.36)	3.0
Copper (mg)	1.69 (0.59–4.32)	1.3
Iodine (μg)	197.00 (55.12–704.40)	200
Selenium (µg)	93.75 (26.26–232.87)	85
Chromium (μg)	35.04 (12.14–103.41)	No recommendation provided
Vitamin A (μg)	823.93 (253.92–3499.41)	1400
Vitamin D (μg)	58.27 (0.67–349.55)	10
Vitamin E (mg)	17.27 (5.70–65.39)	11
Vitamin K (μg)	127.36 (35.60–1088.49)	65
Vitamin C (mg)	151.15 (32.77–1137.24)	155
Vitamin B_1_ (mg)	2.02 (0.52–14.74)	1.2
Vitamin B_2_ (mg)	2.12 (0.70–11.34)	2.0
Niacin (mg)	26.77 (6.17–87.82)	19
Pantothenic acid (mg)	7.47 (2.48–47.75)	7.0
Vitamin B_6_ (mg)	3.07 (0.73–52.99)	1.7
Biotin (μg)	68.72 (17.71–10,244.83)	45
Folate (μg)	434.01 (149.36–1852.19)	490
Vitamin B_12_ (μg)	6.44 (0.87–241.66)	5.5

The following nutrients were most taken as dietary supplements—vitamin C (*n* = 36), calcium (*n* = 37), iron (*n* = 38), omega-3 fatty acids (*n* = 42), and vitamin D (*n* = 54) (Table 7). For some participants, the high nutrient intake was related to the use of dietary supplements, meal replacements, and medications (for example, a very high biotin intake due to the use of medication to treat hair loss).

A significantly higher LNDFH I concentration in human milk was linked to supplemental zinc use (527.10 mg L^−1^ vs. 409.15 mg L^−1^, *p* = 0.033), but a significantly higher 3′-FL concentration in human milk was associated with supplemental omega-3 fatty acids (eicosapentaenoic acid, docosahexaenoic acid) use (1554.33 mg L^−1^ vs. 1167.73 mg L^−1^, *p* = 0.042). A significantly higher 6′-SL concentration in human milk was linked to vitamin D supplemental use (154.00 mg L^−1^ vs. 56.41 mg L^−1^, *p* = 0.014). A weak significant positive association was also found between 6′-SL concentration in human milk and total vitamin D intake (ρ = 0.255, *p* = 0.035), but after adjusting for a covariate—time postpartum—the association was no longer significant (ρ = 0.048, *p* = 0.701). Supplemental calcium intake was associated with a higher 2′-FL, 6′-SL, and LNDFH I concentration in human milk (Table 8). However, total calcium intake was not related to these observed differences in HMO composition (*p* > 0.050).

**Figure 2:**
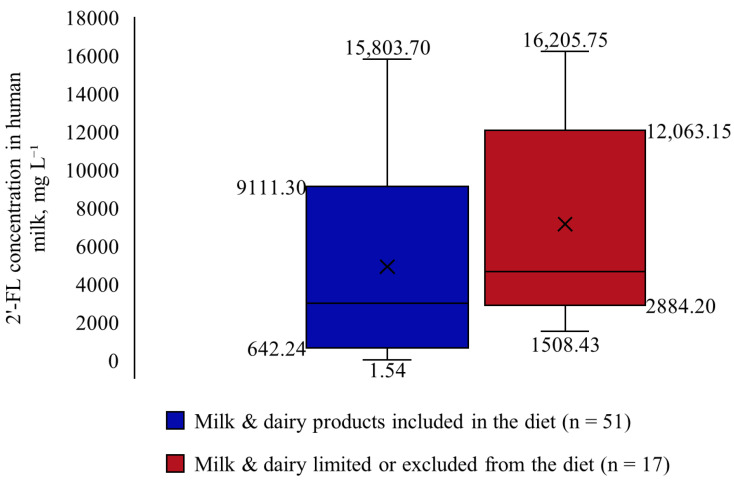
2′-FL concentration in human milk depending on milk and dairy product consumption.

For those participants who had excluded or limited milk and dairy products from their diet, a significantly higher 2′-FL concentration in milk was observed (2970.00 mg L^−1^ vs. 4640.35 mg L^−1^, *p* = 0.037) (Figure 2).

Macronutrient—fat, protein, carbohydrate—intake had no significant impact on qualitative and quantitative HMO composition, except for a weak positive association between protein intake and LNnT (ρ = 0.315, *p* = 0.009). A higher LNnT concentration in human milk was also observed among participants with a higher potassium (ρ = 0.318, *p* = 0.008), phosphorus (ρ = 0.315, *p* = 0.009), and vitamin A intake (ρ = 0.366, *p* = 0.002). However, all associations were no longer significant after adjusting for the covariate—time postpartum (*p* > 0.050 for all).

**Figure 3:**
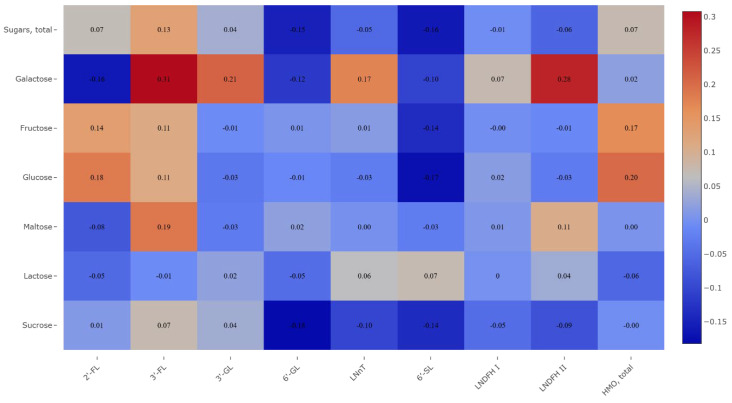
Correlations between HMO concentration in the tested human milk samples and participants’ intake of sugars (*n* = 68).

Overall, sugar intake had no significant effect on HMO concentrations. Only a weak positive association was found between galactose intake and 3′-FL (ρ = 0.308, *p* = 0.011) and LNDFH II (ρ = 0.281, *p* = 0.020) (Figure 3).

A significantly higher HMO concentration in human milk was found among participants who noted that they prefer to consume products where sugar has been replaced with sweeteners (i.e., “zero sugar” products) (Table 9).

## 4. Discussion

This is the first study to report data on HMOs among lactating women in Latvia. 2′-FL was the most prevalent HMO in milk (median concentration in milk—3647 mg L^−1^), which is higher than values reported by other countries [4,26,27]. The 2′-FL concentration of 100 mg L^−1^ in human milk has been suggested as a threshold to phenotypically distinguish mothers by the secretor status [30]. Secretor status is linked with the expression of the secretor gene, which codes for the fucosyltransferase 2 (FUT2) enzyme. Secretor mothers are characterized by the presence of α1,2-fucosylated HMOs in milk (such as 2′FL), while non-secretor mothers produce milk with little to no α1,2-fucosylated HMOs [30]. Eight participants (12%) from this study had a 2′-FL concentration in milk below 100 mg L^−1^, indicating that they are non-secretors. Overall, it is estimated that in Europe, one in five mothers is a non-secretor [31]. However, secretory frequency in Europe is not equal. Around 70–90% of women living in Northern Europe are secretors, while those values are lower (i.e., 50–60%) for women living in the Mediterranean region [32]. Therefore, it is only self-evident that our reported results from the Baltic country of Latvia (88% of participants defined as secretors) are closer to the values reported from Northen Europe.

3′-FL was the second most prevalent HMO in milk among participants in this study (median concentration in milk—1437 mg L^−1^). Its concentration in human milk also varies geographically—a four times higher 3′-FL concentration in human milk has been reported among lactating women from Northern Europe—Sweden (473 nmol mL^−1^)—compared to Africa—rural Gambia (103 nmol mL^−1^) [12].

3′-GL and 6′-GL were detected in human milk analysis in trace amounts. Although the enzymes involved in the synthesis of galacto-oligosaccharides in human milk are not known [33], we assume that microorganisms present in human milk could be responsible for galacto-oligosaccharide synthesis in human milk, similarly to galacto-oligosaccharide formation during milk fermentation with lactic acid bacteria cultures [34].

While the majority of HMOs decline as lactation progresses, certain HMOs—such as 3′-FL, 3′-SL, and Disialyllacto-N-tetraose (DSLNT)—tend to rise in concentration in milk during the early months of lactation [35]. In this study, a moderate negative association was found between 6′-SL concentration in human milk and the infant’s age (ρ = −0.403, *p* = 0.001). A similar observation has reported by McGuire et al. [12].

HMO profile can be influenced by factors such as body mass index, but the data is discrepant. Some studies have reported that BMI may positively correlate with 2′-FL, but negatively with LNnT concentration in human milk [12]. Others report that 2′-FL, LNnT, 3′-SL, and 6′-SL concentrations in human milk were lower for women with overweight or obesity compared to women with normal weight [14]. In our study, the BMI ranged from 16.71 to 33.22, with 29% of participants being overweight or obese (i.e., BMI ≥ 25). This study, like that of Selma-Royo et al. [13], did not find any association between maternal BMI and HMO profiles (*p* > 0.050 for all HMOs). However, calculated BMI based on self-reported data may not be the most appropriate indicator for weight evaluation during the postpartum period. Additional anthropometrical measurements should be performed (like the use of bioelectric impedance analysis scales) [36].

Many studies have reported that maternal diet is associated with the composition and diversity of HMOs [11,13]. However, reported results on which HMOs are affected by specific nutrient intake vary. For example, Selma-Royo et al., 2022 [13] reported that differences in HMO profiles were dependent on maternal secretor status and the nutrient intakes, such as fructose, galactose, fiber, and polyphenols. In this study, we did not observe that fiber intake had a significant impact on HMO composition (*p* > 0.050 for all HMOs), and opposite to Selma-Royo et al.’s [13] observations, we report on a positive association between galactose intake and 3′-FL concentration in human milk (ρ = 0.301, *p* = 0.011).

Consumption of vegetables and fruits or additional intake of vitamins is associated with a higher concentration of fucosylated and sialylated HMOs in human milk [37]. In this study, only a weak positive association was found between LNnT concentration in human milk and potassium, phosphorus, and vitamin A intake. The use of dietary supplements was common among the study participants and was linked to differences in composition and concentration of HMOs in human milk. For example, a threefold higher 6′-SL concentration in human milk was observed among the participants with vitamin D supplemental use (154.00 mg L^−1^ vs. 56.41 mg L^−1^, *p* = 0.014).

There has been growing interest in the potential usefulness of sugar-free sweeteners in reducing sugar intake at the population level, and a variety of “zero sugar” products are now widely available in stores [38]. However, the World Health Organization recommends against the use of non-sugar sweeteners (acesulfame K, aspartame, saccharin, sucralose, stevia and stevia derivatives, etc.) as there may be potential undesirable effects from long-term use, such as an increased risk of type 2 diabetes, cardiovascular diseases, and mortality in adults. This recommendation, however, does not apply to low-calorie sugars and sugar alcohols (polyols) [38]. It has previously been reported that sugar-free sweeteners pass into human milk [39], but it is possible that their intake also potentially affects the HMO composition, as it was found in this study that a preference for “zero sugar” products can significantly impact the 3′-FL, 6′- GL, 6′-SL, LNnT, and LNDFH II concentration in milk (*p* < 0.050 for all above-mentioned HMOs). In the questionnaire, participants were only asked whether they preferred products in which sugar is replaced with sweeteners without any specification of which types of sweetener this might include. Therefore, the participants’ responses likely encompass a broad spectrum of sweeteners that can be used for “zero sugar” products. However, considering that the NutriData program [21] provides information on the polyol content in various products, we were able to evaluate that the amount of polyols consumed had no significant effect on HMO composition and concentration (*p* > 0.050 for all HMOs).

AT and the use of probiotic supplementation may not only affect the maternal microbiome but also potentially impact the HMO composition and concentration in human milk [15]. ATs are among the most frequently prescribed medications during the antepartum, intrapartum, or postpartum period. The reasons for AT are variable and range from urogenital infections to the prophylactic therapy in cases of Group B streptococci, caesarean section prophylaxis, treatment for mastitis, etc. [40]. During this study, almost half of the participants (*n* = 28, 41%) reported receiving AT during the antepartum, intrapartum, and/or postpartum period. However, no significant impact was found regarding the use of AT and HMO composition and concentration in milk (*p* > 0.050 for all HMOs). Prophylactic probiotic use is often prescribed alongside AT to prevent gut microbial diversity changes [41]. In this study, approximately one in five participants (*n* = 15, 22%) reported taking probiotics, but this was not found to significantly affect HMO composition and concentration in milk (*p* > 0.050 for all HMOs).

Smoking and exposure to second-hand smoke result in changes in the composition of human milk, increasing concentrations of heavy metals like cadmium and lead, and decreasing the concentration of essential elements like iodine [42,43]. To our knowledge, currently no published studies show a clear, direct effect of maternal smoking or second-hand smoke on HMO composition in human milk. None of the participants reported current smoking, but 12 participants reported smoking before pregnancy, and for those participants, a higher 6′-SL concentration in human milk was observed. Every fifth participant (*n* = 16, 24%) indicated that a family member smokes, which means that they are potentially exposed to second-hand and third-hand smoke. This factor, however, was not found to significantly affect HMOs’ composition and concentration in milk (*p* > 0.050 for all HMOs). The assessment of smoking impact on human milk composition, however, is complicated, as, in addition to cigarette smoking, the use of other products such as heated tobacco products, e-cigarettes, vapes, and other electronic nicotine delivery systems has become widespread [44]. Given that it is unknown how those novel smoking products affect the composition of human milk, women should be encouraged to refrain from all types of smoking, not only during pregnancy but also during the lactation period. Also, exposure to second-hand and third-hand smoke should be limited as much as possible [45,46].

## 5. Conclusions

This study provides the first Latvian data on HMOs, thereby contributing to the advancement of knowledge on human milk composition across Europe. HMO structural diversity and concentration are closely tied to biosynthesis, as well as maternal factors, including diet, modulating HMO profile in human milk. Overall, this study has identified potential associations between maternal dietary intake and the HMO composition of human milk, though more human milk samples and dietary data need to be evaluated to confirm these relationships and draw further conclusions.

## Figures and Tables

**Table 1:** Gradient separation of human milk oligosaccharides using fluorescence detection.

Time, min	Flow Rate, mL min^−1^	Solvent A, %	Solvent B, %
0.00	0.5000	12.0	88.0
7.00	0.5000	12.0	88.0
17.00	0.5000	15.0	85.0
36.00	0.5000	27.4	72.6
40.50	0.4000	27.4	72.6
41.50	0.4000	30.0	70.0
42.00	0.4000	40.0	60.0
42.50	0.3000	42.0	58.0
45.00	0.3000	70.0	30.0
47.00	0.2000	70.0	30.0
49.50	0.3000	70.0	30.0
52.00	0.3000	12.0	88.0
54.00	0.5000	12.0	88.0
60.00	0.5000	12.0	88.0

**Table 2:** Maternal and infants’ characteristics.

Maternal Characteristics	Median (Minimal–Maximal Value) or Value (*n*, %)
Maternal age (years)	32 (23–40)
Maternal BMI (kg m^2^) ^1^	23.35 (16.71–33.22)
Weight gain during pregnancy (kg)	13 (−8–30)
Nationality	Latvian (*n* = 62, 91%) Other (*n* = 6, 9%)
Populated area	Capital city (*n* = 25, 37%) State city (*n* = 11, 16%) Town (*n* = 13, 19%) Village or farmstead (*n* = 19, 28%)
Educational degree	Upper Secondary (*n* = 4, 6%) College (*n* = 6, 9%) Unfinished Higher (*n* = 6, 9%) Higher (*n* = 52, 76%)
Total household income after tax (EUR)	No information provided (*n* = 6, 9%) ≤1000 (*n* = 3, 4%) ≤2000 (*n* = 14, 21%) ≤3000 (*n* = 25, 37%) >3000 (*n* = 20, 29%)
Mode of delivery	Vaginal (*n* = 52; 76%) Caesarean (*n* = 16; 24%)
Exclusive breastfeeding (months)	4 (1–6)
AT received	No AT received (*n* = 40, 59%) Antepartum (*n* = 8, 12%) Intrapartum (*n* = 16, 24%) Postpartum (*n* = 7, 10%) Not sure if AT received (*n* = 2, 3%)
Chosen human milk sample collection method	By hand (*n* = 21, 31%) Breast pump (*n* = 40, 59%) Both methods (*n* = 7, 10%)
**Infants’ Characteristics**	**Median (Minimal–Maximal Value) or** **Value (*n*, %)**
Sex	Girl (*n* = 33, 49%) Boy (*n* = 35, 51%)
Birth weight (g)	2500–3999 (*n* = 57, 84%) 4000–4499 (*n* = 9, 13%) ≥4500 (*n* = 2, 3%)
Birth length (cm)	53 (48–59)
Colics ^2^	No (*n* = 42, 62%) Yes (*n* = 14, 20%) Not sure (*n* = 12, 18%)

^1^ No anthropometric measurements were performed. BMI was calculated based on the weight and height values reported by participants. ^2^ The following definition for the term “Colics” was provided in the questionnaire: “Inconsolable crying of an infant for more than three hours per day, more than three days per week, for longer than three weeks” [25].

**Table 3:** Human milk composition (%) among the participants (*n* = 68).

Components	Median (Minimal–Maximal Value)	Latvian Study (Median, Range) [16]
Fat	3.32 (1.77–6.60)	4.40 (1.00–7.70)
Protein	1.26 (0.98–1.69)	1.08 (0.75–1.92)
Lactose	6.99 (6.24–7.43)	6.52 (3.29–7.30)
Total solids	11.93 (10.09–15.65)	No data

**Table 7:** Dietary supplement use among the participants (*n* = 68).

Dietary Supplement Use	No	Yes
Folic acid	*n* = 44, 65%	*n* = 24, 35%
Vitamin B_6_	*n* = 42, 62%	*n* = 26, 38%
Other B group vitamins	*n* = 48, 71%	*n* = 20, 29%
Vitamin C	*n* = 32, 47%	*n* = 36, 53%
Vitamin D	*n* = 14, 21%	*n* = 54, 79%
Vitamin E	*n* = 52, 76%	*n* = 16, 24%
Vitamin A	*n* = 53, 78%	*n* = 15, 22%
Vitamin K	*n* = 51, 75%	*n* = 17, 25%
Calcium	*n* = 31, 46%	*n* = 37, 54%
Zinc	*n* = 40, 59%	*n* = 28, 41%
Magnesium	*n* = 38, 56%	*n* = 30, 44%
Iron	*n* = 30, 44%	*n* = 38, 56%
Potassium	*n* = 59, 87%	*n* = 9, 13%
Selenium	*n* = 49, 72%	*n* = 19, 28%
Iodine	*n* = 46, 68%	*n* = 22, 32%
Chromium	*n* = 57, 84%	*n* = 11, 16%
Omega-3 fatty acids (eicosapentaenoic acid, docosahexaenoic acid)	*n* = 26, 38%	*n* = 42, 62%
Other omega fatty acids	*n* = 62, 91%	*n* = 6, 9%
Probiotic supplementation	*n* = 53, 78%	*n* = 15, 22%

**Table 8:** HMO concentration in human milk (mg L^−1^) based on participants’ use of dietary supplement—calcium (*n* = 68).

HMO	No Supplemental Calcium Use (*n* = 31)	Supplemental Calcium Use (*n* = 37)	*p*-Values ^1^
2′-FL	1520.86	5434.55	**0.019**
3′-FL	1367.19	1510.25	0.358
3′-GL	0.00	0.00	0.560
6′- GL	9.24	11.66	0.401
LNnT	214.23	313.50	0.392
6′-SL	81.02	162.36	**0.020**
LNDFH I	350.19	575.14	**0.014**
LNDFH II	138.00	150.26	0.975
Sum of HMOs	4913.70	11,145.20	**0.039**

^1^ Statistically significant values are indicated in bold.

**Table 9:** HMO concentration in human milk (mg L^−1^) based on participants’ preference for “Zero Sugar” products (*n* = 68).

HMO	Preference for “Zero Sugar” Products (*n* = 13)	No Preference for “Zero Sugar” Products (*n* = 55)	*p*-Values ^1^
2′-FL	7123.60	3505.15	0.337
3′-FL	2850.10	1377.75	**0.019**
3′-GL	0.00	0.00	0.312
6′-GL	17.05	8.36	**0.037**
LNnT	613.69	203.67	**0.001**
6′-SL	228.31	111.43	**0.015**
LNDFH I	575.14	470.75	0.149
LNDFH II	236.89	113.96	**0.011**
Sum of HMOs	14,268.34	7731.36	**0.013**

^1^ Statistically significant values are indicated in bold.

## Data Availability

Data supporting reported results are deposited at the Latvian National Research Data Repository Dataverse.lv. https://doi.org/10.71782/DATA/RGML1E (accessed on 28 December 2025).

## Abbreviations

The following abbreviations are used in this manuscript:

2′-FL	2′- Fucosyllactose
3′-FL	3′-Fucolyllactose
3′-GL	3′-Galactosyllactose
6′-GL	6′-Galactosyllactose
6′-SL	6′-Sialyllactose
AT	Antibacterial therapy
BMI	Body mass index
E%	Energy percentage intake
FFQ	Food frequency questionnaire
FLD	Fluorescence detector
HILIC	Hydrophilic interaction liquid chromatography
HMOs	Human milk oligosaccharides
ISO	International Organization for Standardization
LNDFH I	Lacto-N-difucohexaose I
LNDFH II	Lacto-N-difucohexaose II
LNnT	Lacto-N-neotetratose
ND	Not detected
UHLPC/FLD	Ultra-high performance liquid chromatography with fluorescence detector
